# Selecting implementation models, theories, and frameworks in which to integrate intersectional approaches

**DOI:** 10.1186/s12874-022-01682-x

**Published:** 2022-08-04

**Authors:** Justin Presseau, Danielle Kasperavicius, Isabel Braganca Rodrigues, Jessica Braimoh, Andrea Chambers, Cole Etherington, Lora Giangregorio, Jenna C. Gibbs, Anik Giguere, Ian D. Graham, Olena Hankivsky, Alison M. Hoens, Jayna Holroyd-Leduc, Christine Kelly, Julia E. Moore, Matteo Ponzano, Malika Sharma, Kathryn M. Sibley, Sharon Straus

**Affiliations:** 1 Clinical Epidemiology, Ottawa Hospital Research Institute, 501 Smyth Road, Ottawa, ON K1H 8L6 Canada; 2 School of Epidemiology and Public Health, University of Ottawa, Ottawa, Canada; 3 School of Psychology, University of Ottawa, Ottawa, Canada; 4 Knowledge Translation Program, Li Ka Shing Knowledge Institute, St. Michael’s Hospital, Unity Health Toronto, Toronto, Canada; 5 Department of Medicine, GERAS Centre for Aging Research, McMaster University, Hamilton, ON Canada; 6 Department of Social Science, York University, Toronto, ON Canada; 7 Public Health Ontario, Toronto, ON Canada; 8 Department of Anesthesiology and Pain Medicine, University of Ottawa, Ottawa, ON Canada; 9 Department of Kinesiology and Health Sciences, and Schlegel Research Institute for Aging, University of Waterloo, Waterloo, ON Canada; 10 Department of Kinesiology and Physical Education, McGill University, Montreal, QC Canada; 11 Department of Family Medicine and Emergency Medicine, Université Laval, Québec, QC Canada; 12 Melbourne School of Population and Global Health, University of Melbourne, Melbourne, VIC Australia; 13 Department of Physical Therapy, University of British Columbia, Vancouver, BC Canada; 14 Departments of Medicine and Community Health Sciences, University of Calgary, Calgary, AB Canada; 15 Department of Community Health Sciences, University of Manitoba, Winnipeg, MB Canada; 16 The Center for Implementation, Toronto, ON Canada; 17 Division of Infectious Diseases, St. Michael’s Hospital, Unity Health Toronto, Toronto, ON Canada; 18 Department of Medicine, University of Toronto, Toronto, ON Canada; 19 George and Fay Yee Centre for Healthcare Innovation, University of Manitoba, Winnipeg, MB Canada

**Keywords:** Intersectionality, Implementation science, Knowledge translation

## Abstract

**Background:**

Models, theories, and frameworks (MTFs) provide the foundation for a cumulative science of implementation, reflecting a shared, evolving understanding of various facets of implementation. One under-represented aspect in implementation MTFs is how intersecting social factors and systems of power and oppression can shape implementation. There is value in enhancing how MTFs in implementation research and practice account for these intersecting factors. Given the large number of MTFs, we sought to identify exemplar MTFs that represent key implementation phases within which to embed an intersectional perspective.

**Methods:**

We used a five-step process to prioritize MTFs for enhancement with an intersectional lens. We mapped 160 MTFs to three previously prioritized phases of the Knowledge-to-Action (KTA) framework. Next, 17 implementation researchers/practitioners, MTF experts, and intersectionality experts agreed on criteria for prioritizing MTFs within each KTA phase. The experts used a modified Delphi process to agree on an exemplar MTF for each of the three prioritized KTA framework phases. Finally, we reached consensus on the final MTFs and contacted the original MTF developers to confirm MTF versions and explore additional insights.

**Results:**

We agreed on three criteria when prioritizing MTFs: acceptability (mean = 3.20, SD = 0.75), applicability (mean = 3.82, SD = 0.72), and usability (median = 4.00, mean = 3.89, SD = 0.31) of the MTF. The top-rated MTFs were the Iowa Model of Evidence-Based Practice to Promote Quality Care for the ‘Identify the problem’ phase (mean = 4.57, SD = 2.31), the Consolidated Framework for Implementation Research for the ‘Assess barriers/facilitators to knowledge use’ phase (mean = 5.79, SD = 1.12), and the Behaviour Change Wheel for the ‘Select, tailor, implement interventions’ phase (mean = 6.36, SD = 1.08).

**Conclusions:**

Our interdisciplinary team engaged in a rigorous process to reach consensus on MTFs reflecting specific phases of the implementation process and prioritized each to serve as an exemplar in which to embed intersectional approaches. The resulting MTFs correspond with specific phases of the KTA framework, which itself may be useful for those seeking particular MTFs for particular KTA phases. This approach also provides a template for how other implementation MTFs could be similarly considered in the future.

**Trial registration:**

Open Science Framework Registration: osf.io/qgh64.

**Supplementary Information:**

The online version contains supplementary material available at 10.1186/s12874-022-01682-x.

## Background

The annual worldwide investment in biomedical and health research is calculated in the hundreds of billions of dollars and predominantly focuses on discovery, with comparatively less on subsequent dissemination and implementation [1, 2]. As a result, some suggest that health research is ‘all breakthrough, no follow-through’ [3]. Knowledge Translation and Implementation Science (KTIS) emerged to address this by (amongst other activities) developing theory, methods, evidence, and transferable principles to foster a shared understanding of factors driving high quality care and health in patients and the public across jurisdictions.

Models, theories, and frameworks (MTFs) form the foundation for drawing from and developing a cumulative, evidence-informed science [4]. MTFs can also guide implementation intervention developers by outlining phases in the development process, factors to help understand and promote change, and approaches to evaluate and demonstrate change [5]. Among the gaps that have emerged when comparing MTFs is the lack of consideration for race, sex, gender, and other social identities and characteristics, and their intersection with systems of power, privilege, and oppression [6]. Given recent calls for prioritizing health equity in implementation science [7], there is a scientific and moral imperative to consider intersectionality when developing implementation interventions, and importantly, not contribute to intervention-generated inequities.

**Fig. 1:**
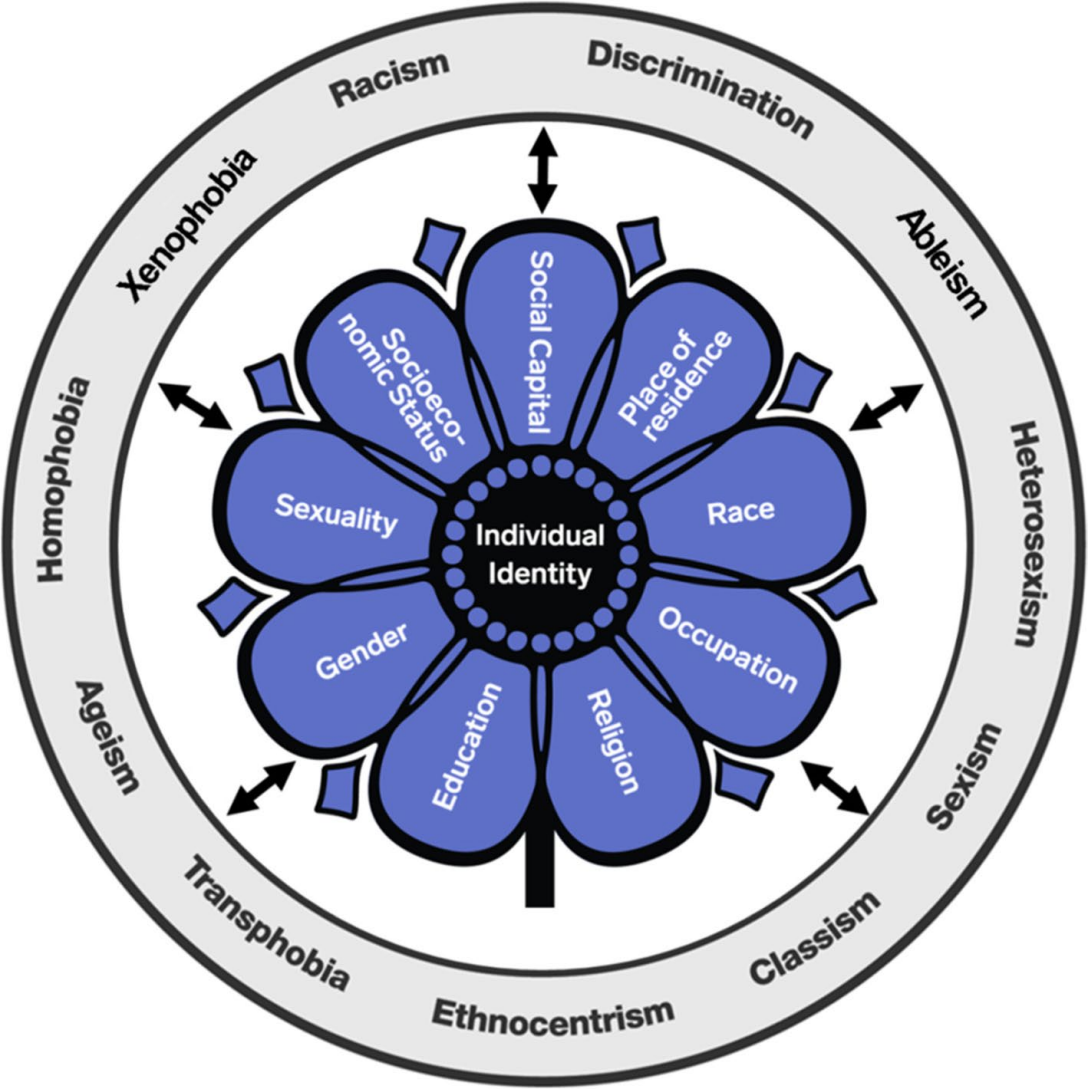
Intersecting individual and contextual factors that can shape individual identity [11, 17]

Intersectionality emerged from women and gender studies, specifically black feminist scholars and activists [8–10]. Intersectionality recognizes that an individual’s experience is shaped by the interaction of factors such as age, disability, place of residence, race, ethnicity, culture, language, occupation, gender and sex, religion, education, socioeconomic status, and social capital [10, 11]. Intersectionality is not only about identity markers, but also about their intersection with systems of power and oppression that result in social hierarchies (e.g., patriarchy, sexism, and racism). While the integration of gender and intersectionality theory into KTIS and health research generally remains in its infancy [1], internationally, funding agencies are increasingly requiring greater consideration for sex and gender-based analyses (SGBA+) [12–15]. Intersectionality highlights not only the importance of sex and gender, but also other core and intersecting factors that shape individuals’ and groups’ identities, experiences, and opportunities (or lack thereof) such as racism, patriarchy, sexism, classism, and colonialism. While individual and contextual factors are captured in equity frameworks [16], intersectionality asserts these should not just be discrete factors in a list of demographics to report in ‘Table 1’ of a manuscript. Rather, intersectionality argues that how these factors interact shapes individual experiences and the constraints or opportunities afforded to them by the systems and structures of power that do or do not cater to those intersecting factors (see Fig. 1). To date, it appears that KTIS MTFs have largely omitted any consideration for how intersecting social identities and social structures could be impeding or supporting the success of a given implementation intervention, or indeed, how they should be considered when developing an implementation intervention. As MTFs form the foundation for a cumulative science, MTFs are a central means for ensuring broad consideration of the role of intersecting factors.

While at face value intersecting factors may seemingly relate most to patients, families, and the public, intersectionality includes a commitment to reflexive practice. As such, there is an opportunity within KTIS and the MTFs in the field to also consider intersectionality from the perspective of those in the healthcare system ultimately tasked with adopting a new practice, changing an existing practice, or setting a policy [6]. Moreover, this lens should be used when considering all stakeholders involved with implementation, including clinicians, managers, and policy makers amongst others.

**Fig. 2:**
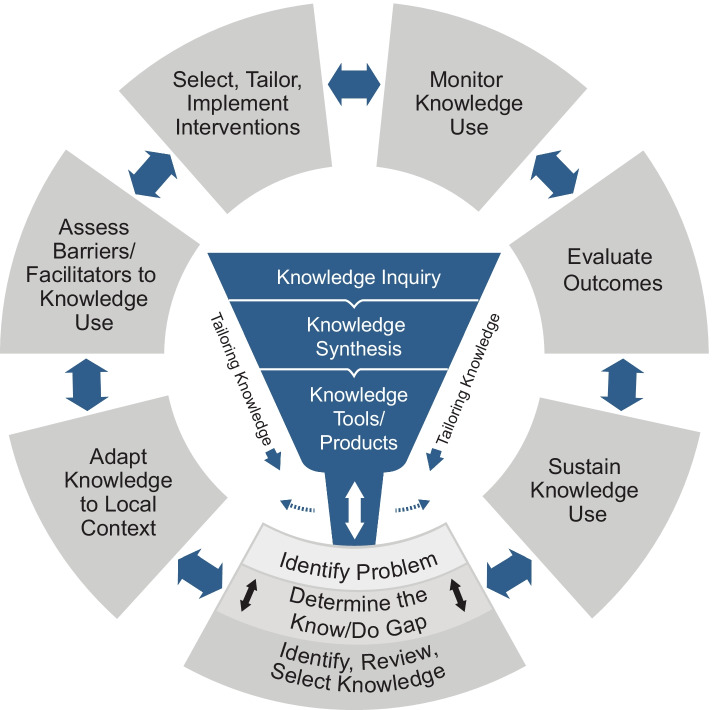
Knowledge-to-Action Framework [18]

The Knowledge-to-Action (KTA; see Fig. 2) framework is a commonly used process model for outlining the phases that KTIS researchers and practitioners can take for implementing evidence into practice [19]. The KTA framework features two key areas of foci, each with specific steps: the ‘knowledge creation funnel,’ which focuses on the conduct and synthesis of primary research to produce an evidence base to inform evidence tools (e.g., clinical practice guidelines, policies); and an ‘action cycle,’ which outlines the key phases for enabling implementation, from identifying evidence-practice gaps to assessing barriers/enablers, tailoring change strategies to these identified barriers/enablers, and monitoring, evaluating, and sustaining change. The KTA framework highlights key phases in the intervention development process upon which more specific MTFs can be applied. For instance, the KTA framework highlights the importance of assessing barriers/enablers but does not describe how to do that. Instead, MTFs that specifically speak to determinants or barriers/enablers to change may be used at that KTA phase. As a synthesis of key process models [19], the KTA framework is a useful set of phases upon which to identify key MTFs to adapt with features of intersectionality for a specific ‘role’ within the wider process of intervention development and evaluation.

While intersectionality can apply to each phase of the KTA process (see Table 1), a challenge for the field is to identify how MTFs used in each phase can be optimized to integrate intersectionality. For example, when assessing barriers and facilitators to knowledge use, a KTIS practitioner can consider how a knowledge user’s location within and experience of the nexus of racism and sexism may impact their behaviour or access (whether that recipient is a patient or citizen, or a healthcare professional applying new guidance in practice). Addressing the challenge requires careful interdisciplinary consideration that includes diverse members of priority populations and those with expertise in KTIS MTFs and intersectionality.

**Table 1:** Intersectionality considerations in the action cycle of the Knowledge-to-Action framework

Knowledge-to-Action Framework Action Cycle Phase	Example Intersectionality Considerations
Identify the Problem	Who says there is a problem? Are they in a position of power? Do oppressed groups also categorize this as a problem?
Adapt Knowledge to Local Context	How can the practice change be adapted to meet practitioner intersections (e.g., age, language, and physical ability)?
Assess Barriers/Facilitators to Knowledge Use	What systems and structures of power contribute to individual-level barriers (e.g., beliefs about one’s capabilities)?
Select, Tailor, Implement Interventions	How can the implementation strategy be tailored to meet patient intersections (e.g., literacy level, language, and racialization)?
Monitor Knowledge Use	Are power dynamics influencing the delivery of the implementation strategy?
Evaluate Outcomes	Are outcomes the same across all patient groups (e.g., racialized immigrant women compared to non-racialized, Canadian-born men)?
Sustain Knowledge Use	Is staff attrition of certain groups (e.g., nurses who are also caregivers during a pandemic) contributing to knowledge loss?

While one approach could involve developing a new MTF, the KTIS literature is replete with existing, well-used, and useful MTFs. To avoid fragmentation of the literature or the need for a new MTF, we argue that it would be more useful to evolve existing MTFs. There are dozens of possible MTFs to choose from [20], and optimizing any existing MTF requires careful development and evaluation. Rather than randomly selecting from amongst the dozens of MTFs available, or preferencing any one MTF simply due to familiarity (cf. Birken et al. [21, 22]), we sought to give all MTFs equal opportunity for consideration. Our objective was to establish consensus on which existing MTFs could represent key implementation phases from the KTA framework and thus serve as exemplars for how intersectionality considerations could be integrated within existing MTFs to inform similar processes for other MTFs. Therefore, we described the interdisciplinary process for prioritizing MTFs, into which we then integrated an intersectional approach.

## Methods

**Fig. 3:**
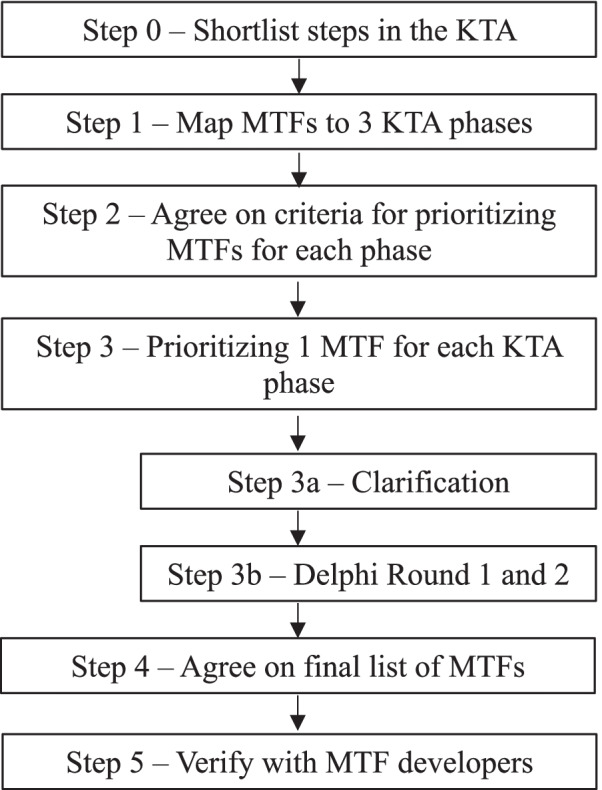
Overview of study

This project involved five steps, described in Fig. 3, and detailed below.

### Project team

Adopting an intersectional approach to research requires ongoing reflexivity, including reflecting on the research team members’ own identities and how these identities may influence our approach. We formed a team in 2016 to respond to a national funding call to understand the impact of gender on Knowledge Translation. We chose to focus our work on KTIS interventions that impacted the lives of older adults. Since 2017, 11 intersectionality scholars, 15 KTIS theorists, 10 KTIS practitioners, and four members of the public (older adults) have been involved in conceiving, planning, and executing project phases. These team members include Project team members who were invited through grant applicants’ circles of contact and recruitment of implementation science or intersectionality scholars and older adults. In February 2020, the thirty-five active team members were sent an anonymous survey to self-report their own intersecting categories. Responses to each question were voluntary; twenty-one team members completed at least one question. Sixteen team members that responded to the survey were between 30 and 87 years old, 24% (4/17 respondents) identified as LGBTQ+, and 94% (15/16 respondents) identified as female. Twenty-two percent (4/18 respondents) reported being part-time employees, students, or retirees; most others (13/18) reported full-time employment. All but one member (17/18) identified as white. Nine out of nineteen respondents (47%) identified having a religious or spiritual affiliation; 85% (17/20 respondents) completed a master’s degree, a doctorate, or professional school. Forty-two percent (8/19 respondents) are the primary caregiver of a child, children, or older adult(s). At the beginning of most project meetings, team members introduced themselves and were asked to reflect on their own position. Recognizing the importance of an interdisciplinary approach for this work, we sought to ensure that members of the research team were oriented to intersectionality, KTIS, MTFs, intervention development, and lived experience of receiving health-promoting interventions. Orientation was provided through a series of webinars, the development of a terms of reference, and ongoing interdisciplinary discussions in person and virtually. Team members voluntarily shared their lived experience at the onset of meetings.

### Step 0: prioritize phases in the KTA framework

The KTA framework includes multiple phases within the inner knowledge creation funnel and the outer action cycle [19]. Our first step was to establish consensus on a subset of phases within the KTA to focus upon for intersectionality enhancement. Prior to the present study and reported elsewhere [23], diverse KTIS experts, KTIS intervention developers and service providers, intersectionality and gender experts, and older adults used a Nominal Group Technique consensus-building approach and prioritized three key phases of the KTA action cycle to evolve using intersectional approaches. The three phases of the KTA selected were: a) identify problem (know-do gap), b) assess barriers and facilitators to knowledge use, and c) select, tailor, and implement interventions [23].

### Step 1: map possible MTFs to KTA phases

Once the three key KTA phases were identified, we used a comprehensive review of KTIS MTFs [20] to identify which among the 159 MTFs identified addressed one or more of the three KTA phases of interest (the KTA framework itself was one of the 160 considered and thus removed). The original papers were sourced for further consideration. Each MTF was then mapped to one or more of the three prioritized KTA phases; 30% were dual mapped independently by two members of the research team with 63% agreement; the remainder were single mapped. Most disagreements were solved by consensus. If an agreement could not be reached, the study’s Principal Investigator (SS) provided a final decision.

### Step 2: agree on criteria for prioritizing MTFs

An interdisciplinary framework committee consisting of 6 KTIS intervention developers, 2 KTIS trainees, 5 MTF experts (i.e., whose research focuses especially on the development and/or application of KTIS MTFs), and 4 with training in intersectionality and critical feminist scholarship developed a survey to determine criteria for prioritizing MTFs. The criteria included in the survey were suggested by committee members and included criteria proposed by the T-CaST theory comparison and selection tool [21]. T-CaST was designed to enable KTIS researchers and practitioners to select, justify, and report the reasons for selecting a given MTF. All committee members were then asked to individually rate the criteria from “1- least important” to “5 - most important” using an online survey (see Additional file 1 for the survey and list of criteria rated). The criteria rated included: whether the MTF: a) is a model, b) is an individual-level behaviour change theory, c) is likely to be familiar to KTIS intervention developers, d) is generalizable to various settings, e) is linked to methods for promoting its use in practice, f) is linked to step-by-step approaches for applying the MTF, g) is likely to be understandable, applicable and can be operationalised by key stakeholders, and h) includes constructs that are relevant to KTIS intervention developers. These were mapped to the T-CAST criteria of ‘usability’, ‘acceptability’ and ‘applicability’, where relevant (see Additional file 1). We held in-person and teleconference discussions, facilitated by JP and DK, on the criteria and survey results to create opportunities for committee members to raise any concerns about the selected criteria. Meeting minutes were circulated following all discussions, and members unable to attend were encouraged to provide feedback to the full group over email or over the telephone with the project coordinator. The discussions centered on how MTFs meet intervention developers’ and users’ needs. The criteria with the highest median ratings and coverage across key T-CaST criteria (usability, acceptability, and applicability) were selected for consideration as the criteria to use when prioritizing MTFs.

### Step 3: prioritizing 1 MTF for each KTA phase

Once the committee agreed on the criteria to use for prioritizing MTFs, smaller groups were formed to consider and prioritize the large number of MTFs.

Step 3a (MTF clarification): Four groups were formed by DK, each including at least one expert in intersectionality, KTIS intervention development, KTIS practice, and KTIS theory. Each group was assigned 33 MTFs, and members were asked to independently review the original article for each in relation to the criteria for prioritizing MTFs. Groups then met in person or online to discuss each MTF in relation to the three prioritized criteria, to clarify conceptual features of the MTFs and to foster interdisciplinary and collaborative decision-making. The discussions considered KTIS practitioners as the target end-users for the MTFs. For example, one group discussed that an MTF focused on learning theory may not be prudent to select as it would only be useful for projects with an educational component. An experienced small group facilitator moderated the discussion, and participants could also share broader comments and questions about the MTFs they reviewed.

Step 3b (Delphi rounds): Members prioritized MTFs for each KTA phase using a modified Delphi approach involving two rounds [24]. In Delphi Round 1, each participant completed an online survey rating the overall importance of each MTF considered in their small group. We used the three criteria agreed upon in Step 2 to define how members should rate ‘overall importance’: MTF’s acceptability, applicability, and usability for KTIS intervention developers. We assumed that all MTFs were compatible with an intersectional lens. We shortlisted MTFs based on the results of Round 1. The shortlist of MTFs were then rated by group members on a 7-point Likert scale ranging from “1 - not at all an important MTF to consider modifying with intersectionality” to “7 - an extremely important MTF to consider modifying with intersectionality”. Medians and interquartile ranges were calculated and MTFs with medians of ≥5 were considered important MTFs for consideration, and these MTFs moved on to Delphi Round 2, which involved the full research team to rate retained MTFs.

Medians and ranges from each small group were then shared with all members of the team, who were given the opportunity to clarify interpretations or voice concerns with not considering a particular MTF (i.e., an MTF with a median < 5). Using a majority vote, participants voted to continue assessing an MTF that had a median of ≥5 in Delphi Round 2. Prior to Delphi Round 2, participants reviewed resources on the top-rated MTFs for each KTA phase to ensure familiarity with the shortlisted MTFs and clarify any concerns, using online resources and teleconferences. Members considered each MTF in relation to each KTA phase that it mapped to (i.e., if an MTF mapped to two KTA phases, the MTF would be considered twice). Participants discussed how acceptable/applicable/usable each MTF was in relation to each prioritized KTA phase to which it was relevant.

In Delphi Round 2, all team members were provided with the list of retained MTFs by KTA phase, the mean and median, and if relevant, their own previous score (if the MTF had been allocated to their small group). A citation search in Google Scholar and PubMed was conducted by a research coordinator in Round 2 to further inform judgements about modifying MTFs with an intersectionality lens; in particular, judgements about whether the MTF is likely to be familiar to KTIS intervention developers. Participants completed an online survey to rate shortlisted MTFs on a 7-point Likert scale ranging from “1 - not at all an important MTF to consider modifying with intersectionality” to “7 - an extremely important MTF to consider modifying with intersectionality” by all participants. Mean scores and standard deviations were calculated (means were preferred at this stage to enable greater granularity). MTFs with mean of ≥5 were considered important MTFs for consideration and moved forward to the final round of rating. Once survey results were presented for each Delphi phase, members were asked “Are there any models/theories/frameworks that you have serious concerns about eliminating at this point?” Responses could be provided within the meeting or by follow-up email to DK. The survey for Delphi Round 2 also contained an open-text box where participants could provide additional considerations or reflections on the MTF.

Results (mean, standard deviation, IQR, median) of the top-rated MTFs for each KTA phase were reviewed with the group via teleconference. Participants were given the opportunity to voice any concerns with not considering a particular MTF (i.e., an MTF with a mean rating < 6) at this stage. Using a majority vote (online), the participants voted on whether to continue assessing an MTF that had a mean rating of < 6 out of 7. Participants then discussed the top-rated MTFs. Using a majority vote, participants decided whether a final rating survey was needed to identify one MTF for each of the three KTA phases. If multiple MTFs had similarly high mean ratings, the members used a majority vote to determine which MTF would be enhanced with intersectionality.

### Step 4: agree on final list of MTFs

One final, majority vote was conducted anonymously through the videoconferencing software (WebEx Chatbox); each member was asked if they agreed (yes/no) on the top-rated MTF for each KTA phase (based on mean scores), with a majority vote.

### Step 5: verify with MTF developers

Following the selection of three MTFs, we contacted the original developers of each MTF to ensure that we were using the most updated versions of the MTFs, gauged their interest in supporting the enhancement for intersectionality, asked about insights to the MTF enhancement process, and inquired about potential dissemination opportunities.

## Results

### Step 1: map possible MTFs to KTA phases

Of the 160 MTFs considered, 12 were removed as they were too specific to be generalized to multiple types of KT interventions or clinical contexts, and two were removed as they were duplicates of other MTFs. For duplicate MTFs with multiple names, the original citation (least recent) was selected. The KTA itself was not considered because it served as the larger overarching framework for this work. Original papers or primary studies could not be retrieved for 11 MTFs and were also excluded. The result was 134 candidate MTFs that described one or more of the three KTA phases prioritized in Step 0, to be considered for enhancement with intersectionality (see Additional file 2).

### Step 2: agree on criteria for prioritizing MTFs

A total of 16 of 17 team members responded to the online survey to agree on which criteria to use for prioritizing MTFs (Additional file 1). The criterion of “ease of enhancing with intersectionality” was not considered as the group assumed that in principle all MTFs were likely amenable to enhancement with intersectionality. However, the group discussed this assumption that all KTIS MTFs could be operationalized with an intersectional lens. Committee members with training in intersectionality and critical feminist scholarship outlined that some MTFs carry a deductive rather than a more narrative style of theory.

The final criteria for judging the priority of an MTF were a) Acceptability (MTF is likely to be familiar to KT intervention developers); b) Applicability (MTF can likely be generalized by KT intervention developers to different populations, settings, and disciplines as needed); and c) Usability (KT intervention developers are likely to be able to understand and operationalize the MTF for the KTA phase under consideration). These criteria were used in subsequent steps to prioritize which MTF could be enhanced with an intersectionality approach for each of the three KTA phases.

### Step 3: prioritizing 1 MTF for each KTA phase

Sixteen of the 17 team members responded to Delphi Round 1 across four small groups. Table 2 summarizes the MTFs retained for each KTA phase from Delphi Round 2. The top-rated MTFs for the Identify the problem phase were the Iowa Model of Evidence-Based Practice to Promote Quality Care (mean = 4.57, SD = 2.31) and the Knowledge Exchange-Decision Support (mean = 4.21, SD = 1.76). The top-rated MTFs for the Assess barriers/facilitators to knowledge use phase include the Consolidated Framework for Implementation Research (CFIR) [25, 26] (mean = 5.79, SD = 1.12) and the Theoretical Domains Framework (TDF) [27] (mean = 5.71, SD = 1.90). The highly rated MTFs for the Select, tailor, implement interventions phase include the Behaviour Change Wheel [28] (mean = 6.36, SD = 1.08) and the CFIR (mean = 5.79, SD = 1.31).

**Table 2:** List of retained MTFs by KTA phase across all groups following Delphi-Round 2

Model/Theory/Framework	Mean	Standard Deviation
**KTA Phase: Identify Problem**		
Iowa Model of Evidence-Based Practice to Promote Quality Care	4.57	2.31
Knowledge Exchange-Decision Support	4.21	1.76
Conceptual framework for context-based evidence-based decision-making	3.86	1.92
Intervention Mapping Framework	3.71	1.98
Promoting Action on Research in Health Services framework	3.64	1.74
Quality Implementation Framework	3.50	1.65
Ecological Framework	3.36	1.69
PRECEDE-PROCEED	3.29	1.73
Organizational Readiness to Change Theory	3.21	1.53
Social Cognitive Theory	3.07	1.49
Organizational Development Theory	3.00	1.80
Organizational Theory of Implementation Effectiveness	2.93	1.38
**KTA Phase: Assess barriers/facilitators to knowledge use**		
Consolidated Framework for Implementation Research (CFIR)	5.79	1.12
Theoretical Domains Framework (TDF)	5.71	1.90
Behaviour Change Wheel	5.57	2.10
Ecological Framework	4.14	1.88
Knowledge Exchange-Decision Support	4.07	1.73
Promoting Action on Research in Health Services framework	4.00	1.71
Plan-Do-Study-Act (PDSA) Cycles	4.00	2.00
Theory of Planned Behavior	4.00	1.75
Social Cognitive Theory	3.86	1.51
Intervention Mapping Framework	3.86	1.70
Health Action Process Approach (HAPA)	3.71	1.98
PRECEDE-PROCEED	3.57	1.70
Conceptual framework for context-based evidence-based decision-making	3.50	2.03
Quality Implementation Framework	3.50	1.51
Organizational Development Theory	3.43	2.03
Iowa Model of Evidence-Based Practice to Promote Quality Care	3.36	1.95
Organizational Theory of Implementation Effectiveness	3.36	1.60
Organizational Readiness to Change Theory	3.00	1.71
**KTA Phase: Select, tailor, implement interventions**		
Behaviour Change Wheel (BCW)	6.36	1.08
Consolidated Framework for Implementation Research (CFIR)	5.79	1.31
Theoretical Domains Framework (TDF)	4.71	2.16
Intervention Mapping Framework	4.57	1.83
Promoting Action on Research in Health Services framework	4.57	1.22
Quality Implementation Framework	4.57	1.50
Plan-Do-Study-Act (PDSA) Cycles	4.29	2.09
Diffusion of Innovations	4.00	1.92
Ecological Framework	3.93	1.77
PRECEDE-PROCEED	3.79	1.97
Health Action Process Approach (HAPA)	3.79	2.01
Social Cognitive Theory	3.71	1.44
Theory of Planned Behavior	3.57	1.60
Iowa Model of Evidence-Based Practice to Promote Quality Care	3.43	2.03
Organizational Development Theory	3.29	1.94
Organizational Theory of Implementation Effectiveness	3.21	1.67
Conceptual framework for context-based evidence-based decision-making	3.21	1.76
Organizational Readiness to Change Theory	3.00	1.71

### Step 4: agree on final list of MTFs

No members voiced issues with the final selected key MTFs to take forward as exemplars. For the Identify the problem phase, we prioritized the Iowa Model of Evidence-based Practice to Promote Quality of Care. For the Assess barriers/facilitators to knowledge use phase, we prioritized the Consolidated Framework for Implementation Research, and for the Select, tailor, implement interventions phase, we selected the Behaviour Change Wheel, including Theoretical Domains Framework.

### Step 5: Verify with MTF developers

All three MTF developers were contacted by the Principal Investigator in November 2018 and informed about their MTF’s selection for the project. Two of three developers responded and confirmed that we were working off the most updated versions and stated that they were interested in seeing the results. No additional insights were provided.

## Discussion

Our interdisciplinary team aimed to rigorously identify MTFs to enhance with intersectionality, rooted in the key phases of the implementation intervention development process described by the KTA framework. The result of this process considered 160 MTFs in detail and prioritized 3 MTFs using agreed criteria of acceptability, applicability, and usability. The resulting MTFs correspond with specific phases of the KTA framework, which itself may be useful for those seeking particular MTFs for particular KTA phases. Namely, using a transparent, interdisciplinary process (see Fig. 3), we reached consensus on the Iowa Model of Evidence-Based Practice to represent the Identify the problem phase, the Consolidated Framework for Implementation Research to represent the Assess barriers/facilitators to knowledge use phase, and the Behaviour Change Wheel (including TDF) [21] as an exemplar of the Select, tailor, implement interventions phase. Each MTF has since been enhanced with intersectional considerations and an accompanying suite of tools to support KTIS researchers and practitioners to apply, use, and pilot test these tools in their delivery of KTIS interventions [29].

The three MTFs selected should be viewed as exemplars. Other KTIS MTFs could and should also be enhanced with intersectionality. However, to maintain feasibility and ensure each of the three MTFs were carefully optimized, we endeavored to ‘start somewhere’ and the presently reported study outlines the process through which we sought to identify those initial three MTFs exemplars. The enhancement of MTFs with intersectionality requires the collective effort of the field to ensure they are enhanced, used, and tested. We took the position that with 160+ MTFs, we do not necessarily need a 161st MTF but rather we should aim to optimize the MTFs that we have and that are the most useful. Thus, in addition to the three selected herein, we hope that others may also work to enhance MTFs of particular use in their setting, using our future enhanced MTFs as examples. In addition, a similar process can be used for prioritizing and enhancing MTFs in other areas of the KTA (including knowledge creation funnel) drawing upon the cross-cutting enhancements made to the exemplar MTFs [30]. That said, if/when new MTFs are developed, the steps outlined herein may also provide a useful starting point for ensuring intersectional approaches are considered (see also Table 3 for suggested steps for enhancing MTFs with an intersectional lens).

**Table 3:** Suggested steps for enhancing MTFs with an intersectional lens

1. Form an interdisciplinary team that contains end users, practitioners, and theorists from a range of backgrounds. 2. Reflect individually and as a group on privilege, oppression, biases, and unique perspectives. 3. Conduct capacity building on definitions and key terms used across disciplines. 4. If applicable, prioritize MTF areas or types of MTFs to modify (e.g., Evaluate Outcomes phase of the KTA model). 5. Decide as a group on what criteria to use for prioritizing the MTFs. 6. Collate a list of all MTFs to consider. • Search for and review MTF syntheses • Probe team members for MTF suggestions 7. Facilitate each team member’s review of the MTF. • Create space for all team members to clarify their understanding of the MTFs • If the list of MTFs is too long, split the team up into smaller interdisciplinary groups 8. Use the Delphi procedure to prioritize an MTF using established criteria. 9. Reach out to the original MTF author to confirm the most updated version of the MTF. 10. Using an iterative approach, work as a team to enhance the MTF with an intersectional lens. Enhancements to consider include: • Apply reflection prompts to model stages. • Re-conceptualize existing MTF constructs to consider broader systems and structures of power. • Use illustrative examples that encapsulate lived experience of intervention recipients. 11. Usability test MTFs with end-users. Modify MTFs accordingly.	

### Challenges, limitations, and opportunities

Parsing and prioritizing from amongst 160 MTFs is not without its challenges, and while we strove to be as transparent and interdisciplinary as possible, the reality of the MTF literature is such that familiarity and clarity of the source papers may have influenced the selection process. Our approach to prioritizing one MTF for each of three KTA phases may implicitly give the impression that the final prioritized MTF for a given KTA phase only applies to that phase. However, that is not necessarily the case; for example, the Behaviour Change Wheel (with TDF) can also be used in earlier phases of the KTA. Nevertheless, by creating small groups and carefully reading through all key papers for each MTF, we endeavored to be as rigorous as possible. While it is possible that a group of different interdisciplinary researchers and practitioners may have prioritized a different set of MTFs, the three selected provide a defensible starting point. We do not see that as a limitation per se and encourage other teams to take this approach with remaining MTFs as this can only serve to further enhance the degree to which MTFs and their applications consider the implications of intersectionality.

One of the goals of intersectionality is to dismantle various forms of oppression and inequality that operate together. To fully do this requires a consideration of how our own methodological approach to intersectionality was also imbued with power. While MTFs were intentionally integrated with an intersectional lens, it was impossible to attend to all possible identities and structures simultaneously; choices were made about which axes of difference and forms of inequality to focus on. Relatedly, at times we may have provisionally used social categories in our intersectional approach that did not challenge assumptions underlying the categorization of social identities (e.g., cisnormativity embedded in the social categories of ‘men’ and ‘women’) [31].

Working with MTFs, the KTIS process, and intersectionality can be challenging but we strove to build capacity across all team members, and this began from the outset of developing the team itself. We took an intersectional approach to recruiting, supporting, and engaging a diverse team and recognized the importance of capacity building in multiple areas before bringing a new lens to MTFs. However, the team was still made up of limited identified intersections, with 94% identifying as female and 94% identifying as white. For example, we engaged in multiple onboarding teleconference meetings with intersectionality experts who at the time were not yet familiar with KTIS. Furthermore, intersectionality experts led webinars and discussions with those on the team with a KTIS background. Within our desire for capacity building and interdisciplinarity, we also recognized, discussed, and accepted that the epistemological foundations of different fields may not always be aligned, yet exploring tensions [32] is necessary for enhancing MTFs. We moved forward under the assumption that all MTFs (despite some challenging tensions in epistemology) could be enhanced with an intersectional lens in a way that advances KTIS. Nevertheless, there are many possible intersecting categories and identities, and future research should continue to explore and apply MTFs across a range of characteristics and their intersection with systems of power and oppression. A full project limitations statement can be found in Additional file 3.

Any proposed changes to MTFs should do justice to the source material while also carefully considering the range of ways in which intersectionality might be considered and integrated. The process must be inherently interdisciplinary and presents both a challenge and opportunity as striving towards interdisciplinary teams involves bringing together expertise that may have divergent epistemological foundations [33]. However, in KTIS, the goal is a shared understanding of how to move evidence into routine practice to improve the health and well-being of citizens globally, and thus any epistemological challenges may indeed rather serve as opportunities to challenge assumptions and broaden perspectives. Further, if the value of MTFs is indeed partly to summarize what we know about a given phenomenon to ensure a cumulative science, there is a real opportunity for advancing the science by carefully integrating and testing propositions inherent to intersectional approaches. Doing so likely also requires adopting an intersectional approach to building KTIS research teams, knowledge user panels, and ultimately, successful integrated KT.

## Conclusions

We rigorously selected three useable, acceptable, and applicable MTFs to enhance with intersectional approaches, each representing a specific phase of the KTA framework for intersectionality enhancement: the Iowa Model of Evidence-Based Practice (for the Identify the problem phase of the KTA), the Consolidated Framework for Implementation Research (for the Assess barriers/facilitators to knowledge use phase), and the Behaviour Change Wheel (for the Select, tailor, implement interventions phase). Whether trying to change what healthcare providers do or supporting patients and the public to achieve greater health, enhancing MTFs and their applications with intersectional approaches will serve to ensure more broadly applicable KTIS interventions that consider intersectional factors. As a field, knowledge translation and implementation science can no longer ignore intersectional factors: it is time to move towards creating teams that bring diverse individuals together to develop KTIS interventions informed by MTFs that account for intersectionality.

## Supplementary Information

**Additional file 1.** Criteria Prioritization Survey and Results.

**Additional file 2.** MTFs selected for consideration.

**Additional file 3.** Project Limitations Statement.

**Additional file 4.** Criteria Prioritization Survey - Models, Theories and Frameworks (MTF).

**Additional file 5.** Intersecting Categories Survey.

**Additional file 6.** Intersecting Categories Survey Results - Project Team.

## Data Availability

All data generated or analyzed during this study are included in this published article [and its supplementary information files].

## Abbreviations

CFIR: Consolidated Framework for Implementation Research
KTA: Knowledge-to-Action
KTIS: Knowledge Translation and Implementation Science
MTFs: models, theories, and frameworks
TDF: Theoretical Domains Framework
