# Supplementary material for: Selecting implementation models, theories, and frameworks in which to integrate intersectional approaches
Source: BMC Med Res Methodol. 2022 Aug 4;22:212. doi: 10.1186/s12874-022-01682-x (PMC9351159; doi:10.1186/s12874-022-01682-x)
Supplement: Supplementary file 1 — Additional file 1. Criteria Prioritization Survey and Results. [file 12874_2022_1682_MOESM1_ESM.docx]

**Criteria Prioritization Survey and Results**

Thank you for all your contributions to the Framework Committee!

As a reminder:

The objective of this research study is for KT intervention developers who are addressing the needs of older adults to use an intersectional approach when designing and implementing interventions.

The role of the Framework Committee is to select and enhance 3 KT MTFs (1 for each of the previously prioritized Knowledge-to-Action Cycle stages) with intersectional approaches.

For your reference:

Please visit work done by (30) for additional information on intersectionality and intersectional approaches

We look to the work of [Nilsen (2015)](about:blank) (5) to define models, theories, and frameworks (MTFs):​

Model - Specifies steps in the process of translating research into practice

Theory - Describes a prediction and causal mechanisms

Framework - Explains factors that influence implementation and outcomes

The list of KT Models/Theories/Frameworks (MTF) that we are selecting from is sourced from [Strifler's (2018)](about:blank) Review(18).

Please see Appendix E of Strifler's Review (also available on the Dropbox) for the full list of KT MTFs under consideration

Survey Part 1: Criteria Prioritization

In order to select which criteria we will use to prioritize which KT MTFs to modify with an intersectional approach, kindly complete the following rating question.

The criteria below are considered feasible and were suggested by members of the Framework Committee.

When considering these criteria, please keep in mind that the audience of the intersectionality-enhanced MTFs is KT intervention developers.

Kindly rate the criteria below from least important (1) to most important (5).

Kindly rate the criteria below from least important (1) to most important (5).

| - MTF is not a model  According to [Nilsen (2015)](about:blank) (5), models specify steps (stages, phases) in the process of translating research into practice. The Knowledge-to-Action Cycle is a process model and is the basis of this project.  As the Framework Committee’s work is based on a model (the Knowlede-to-Action Cycle, this criterion prompts Framework Committee members to consider that it may be redundant to select another KT model to enhance with intersectionality.   This criterion has been mapped for all of the MTFs in Strifler's review. |
| --- |
| - MTF is not an individual-level behaviour change theory  According to [Nilsen (2015)](about:blank) (5), theories lay out a causal mechanism of how something happens. Individual behaviour change theories outline how an individual changes their behaviour.   This criterion prompts Framework Committee members to consider whether theories developed to understand how individuals change their behaviour are relevant to the work of KT Intervention Developers.  This criterion has been mapped for all of the MTFs in Strifler's Review. |
| - MTF is likely to be familiar to target audience (KT Intervention Developers)   This criterion prompts consideration of how familiar the population of KT Intervention Developers are with the MTF.   To the best of their ability, this criterion would be assessed by Framework Committee members in their review of MTFs. Additional research may be required. |
| - Generalizability of MTF for target audience (KT Intervention Developers)  As KT Intervention Developers work in a variety of settings, this criterion prompts consideration that the MTFs selected should be applicable and generalizable to various disciplines (e.g., health services, social work) and settings (e.g., long term care homes, hospitals).  To measure applicability, Framework Committee members will consider whether the MTF could be used in >3 disciplines and > 3 settings. |
| - MTF authors provide methods for promoting its implementation in practice  This criterion prompts consideration of whether the MTF has explicit methods for promoting its use by KT Intervention Developers.  To the best of their ability, this criterion would be assessed by Framework Committee members in their review of MTFs. Additional research will be required to investigate and source the existence of methods for implementing each MTF. |
| - MTF authors provides a step-by-step approach for applying it  This criterion prompts consideration of whether there are step-by-step instructions that outline how an MTF can be applied.  To the best of their ability, this criterion would be assessed by Framework Committee members in their review of MTFs. Additional research will be required to investigate and source the existence of step-by-step instructions for applying MTFs. |
| - Key stakeholders (i.e., KT Intervention Developers) are likely able to understand, apply, and operationalize MTF  This criterion prompts consideration of how usable the MTF is for KT Intervention Developers.  To the best of their ability, This criterion would be assessed by Framework Committee members in their review of MTFs. |
| - MTF includes relevant constructs for KT Intervention Developers  This criterion prompts consideration of whether the MTF contains key constructs for the target audience.  To the best of their ability, this criterion would be assessed by Framework Committee members in their review of MTFs. |

Additional Table 1. Results of prioritized criteria rating

| Potential Criteria for prioritizing MTFs | Agreement (1 strongly disagree to use – 5 strongly agree to use) | | |
| --- | --- | --- | --- |
|  | Median | Mean | Standard Deviation |
| MTF is not a model | 3.00 | 2.82 | 0.57 |
| MTF is not an individual-level behaviour change theory | 2.50 | 2.75 | 0.83 |
| Acceptability: MTF is likely to be familiar to KT Intervention Developers | 3.00 | 3.20 | 0.75 |
| Applicability: Generalizability of MTF to KT Intervention Developers | 4.00 | 3.82 | 0.72 |
| Usability: MTF authors provide methods for promoting its implementation in practice | 3.00 | 3.43 | 0.73 |
| Usability: MTF authors provide a step-by-step approach for applying it | 3.00 | 2.93 | 0.96 |
| Usability: Key Stakeholders (i.e., KT Intervention Developers) are able to understand and operationalize MTF | 4.00 | 3.89 | 0.31 |
| Usability: MTF includes relevant constructs for KT Intervention Developers | 3.00 | 3.00 | 0.82 |
