# Supplementary material for: Selecting implementation models, theories, and frameworks in which to integrate intersectional approaches
Source: BMC Med Res Methodol. 2022 Aug 4;22:212. doi: 10.1186/s12874-022-01682-x (PMC9351159; doi:10.1186/s12874-022-01682-x)
Supplement: Supplementary file 2 — Additional file 2. MTFs selected for consideration. [file 12874_2022_1682_MOESM2_ESM.docx]

MTFs selected for consideration

| **KT Model/Theory/Framework (Strifler et al. 2018 Appendix E)** | **Relevant KTA Cycle Stage (1,3, and/or 4)** | **Notes** |
| --- | --- | --- |
| Clinical Work Assessment Model | Stages 1 & 3 |  |
| Knowledge Exchange-Decision Support (Kazanjian et al.) | Stages 1 & 3 |  |
| Social Cognitive Theory (Bandura) | Stages 1 & 3 |  |
| Toward Evidence-Informed Practice Program Evidence Tool (Ontario Public Health Association) | Stages 1 & 4 |  |
| A Conceptual Framework of Interaction and Research Utilization | Stages 1 & 3 & 4 |  |
| Action Research | Stages 1 & 3 & 4 |  |
| Active Implementation Framework | Stages 1 & 3 & 4 | Not contained in Strifler et al. - added by Framework Committee Member |
| Adult Learning Principles (Vella) | Stages 1 & 3 & 4 |  |
| Adult Learning Theory (Knowles) | Stages 1 & 3 & 4 |  |
| CAN-IMPLEMENT (Canadian Guideline Adaption Study Group) | Stages 1 & 3 & 4 |  |
| Checkland's Soft Systems Methodology (Checkland) | Stages 1 & 3 & 4 |  |
| Collaborative Model for Achieving Breakthrough Improvement (Institute for Healthcare Improvement) | Stages 1 & 3 & 4 |  |
| Community of Practice Theory | Stages 1 & 3 & 4 |  |
| Community Organization and Development for Health Promotion Model (Braithwaite et al.) | Stages 1 & 3 & 4 |  |
| Complex Adaptive Systems | Stages 1 & 3 & 4 |  |
| Complexity theory | Stages 1 & 3 & 4 |  |
| Conceptual framework for context-based evidence-based decision-making | Stages 1 & 3 & 4 |  |
| Conduct and Utilization of Research in Nursing Project Model (Horsley et al.) | Stages 1 & 3 & 4 |  |
| Ecological Framework (Sallis & Owen) | Stages 1 & 3 & 4 |  |
| Ecological Model of Health Behaviour (McLeroy et al.) | Stages 1 & 3 & 4 |  |
| Empowerment Pedagogy (Freire) | Stages 1 & 3 & 4 |  |
| Factors Determining Rate of Adoption of Research Innovations into Practice (Bradley et al.) | Stages 1 & 3 & 4 |  |
| Implementation Process Model (Brand) | Stages 1 & 3 & 4 |  |
| Interactive Systems Framework (Wandersman) | Stages 1 & 3 & 4 |  |
| Intervention Mapping Framework (Bartholomew et al.) | Stages 1 & 3 & 4 |  |
| Iowa Model of Evidence-Based Practice to Promote Quality Care (Titler) | Stages 1 & 3 & 4 |  |
| LEAN transformation process | Stages 1 & 3 & 4 |  |
| Learning theory | Stages 1 & 3 & 4 |  |
| Navigation Chart (Kubina & Kelly) | Stages 1 & 3 & 4 |  |
| Organizational Development Theory | Stages 1 & 3 & 4 |  |
| Organizational Readiness to Change Theory (Weiner) | Stages 1 & 3 & 4 |  |
| Organizational Theory of Implementation Effectiveness (Weiner) | Stages 1 & 3 & 4 |  |
| Ottawa decision support framework | Stages 1 & 3 & 4 |  |
| Ottawa Model of Research Use (Graham & Logan) | Stages 1 & 3 & 4 |  |
| Political Economy of Health (Minkler et al.) | Stages 1 & 3 & 4 |  |
| PRECEDE-PROCEED (Green) | Stages 1 & 3 & 4 |  |
| Promoting Action on Research in Health Services framework | Stages 1 & 3 & 4 |  |
| Promoting Action on Research Implementation in Health Services (Kitson et al.) | Stages 1 & 3 & 4 |  |
| Push-Pull-Infrastructure Model | Stages 1 & 3 & 4 |  |
| Quality Implementation Framework (Meyers, Durlak & Wandersman) | Stages 1 & 3 & 4 |  |
| Reach Effectiveness Adoption Implementation Maintenance (RE-AIM) (Glasgow et al.) | Stages 1 & 3 & 4 |  |
| Regulative Research Cycle (van Strien) | Stages 1 & 3 & 4 |  |
| Research and Evidence in Practice Model (Caldwell et al.) | Stages 1 & 3 & 4 |  |
| Research and Policy in International Development (RAPID) framework | Stages 1 & 3 & 4 |  |
| Situated Learning Theory (Lave & Wenger) | Stages 1 & 3 & 4 |  |
| Social Change Theory (Thompson & Kinne) | Stages 1 & 3 & 4 |  |
| Social Ecological Model (Bronfenbrenner) | Stages 1 & 3 & 4 |  |
| Social Ecological Model for Health Promotion (Stokols) | Stages 1 & 3 & 4 |  |
| Social Learning Theory (Bandura) | Stages 1 & 3 & 4 |  |
| Social network theory | Stages 1 & 3 & 4 |  |
| Stage theory of organizational change | Stages 1 & 3 & 4 |  |
| Structural Ecological Model (Cohen et al.) | Stages 1 & 3 & 4 |  |
| Systems theory | Stages 1 & 3 & 4 |  |
| Ten Steps to Systems Thinking (WHO) | Stages 1 & 3 & 4 |  |
| Theory of ethical space | Stages 1 & 3 & 4 |  |
| Theories of Change Evaluation Model (Connel & Kubisch) | Stages 1 & 3 & 4 |  |
| Three-World View Model (Peek) | Stages 1 & 3 & 4 |  |
| Taxonomy of Critical Success Factors (Williams & Ramaprasad) | Stage 3 |  |
| Behavioural Choice Theory (Epstein) | Stage 3 |  |
| Health Empowerment Theory (Shearer) | Stage 3 |  |
| Strengths Weaknesses Opportunities Threats (SWOT) | Stage 3 |  |
| Prospect Theory (Kahneman & Tversky) | Stages 3 & 4 |  |
| 4MAT System (McCarthy) | Stages 3 & 4 |  |
| 5 A’s Model (Goldstein et al.) | Stages 3 & 4 |  |
| Advancing Research and Clinical practice through close Collaboration Model (Melnyk & Fineout-Overholt) | Stages 3 & 4 |  |
| Ask, assist, refer clinical action framework | Stages 3 & 4 |  |
| Attitude-Social Influence-Self-Efficacy (ASE) Model (De Vries et al.) | Stages 3 & 4 |  |
| Awareness to Adherence Model (Pathman et al.) | Stages 3 & 4 |  |
| Behaviour Change Wheel (Michie) | Stages 3 & 4 |  |
| Behavioural Alternative Model (Jaccard) | Stages 3 & 4 |  |
| Brief action planning | Stages 3 & 4 |  |
| Capability Opportunity Motivation and Behaviour (COM-B) (Michie) | Stages 3 & 4 |  |
| Cognitive behavioural theory | Stages 3 & 4 |  |
| Cognitive Theory (Lewin) | Stages 3 & 4 |  |
| Cognitive-Social Health Information-Processing (C-SHIP) Model | Stages 3 & 4 |  |
| Communication-Behaviour Change Model (McGuire) | Stages 3 & 4 |  |
| Communications theory | Stages 3 & 4 |  |
| Community Coalition Action Theory | Stages 3 & 4 |  |
| Community Connection Model (Liddy et al.) | Stages 3 & 4 |  |
| Complex Innovations Implementation Framework (Helfrich et al.) | Stages 3 & 4 |  |
| Conceptual Framework for Addressing Social Context of Health Behaviours (Sorensen) | Stages 3 & 4 |  |
| Conceptual Model (Lara et al.) | Stages 3 & 4 |  |
| Consolidated Framework for Implementation Research (CFIR) (Damschroder) | Stages 3 & 4 |  |
| Constructivist Learning Theory (Phillips) | Stages 3 & 4 |  |
| Control Theory (Carver & Scheier) | Stages 3 & 4 |  |
| Elaboration Likelihood Model (Petty & Cacioppo) | Stages 3 & 4 |  |
| Expectancy-Value Motivation Theory | Stages 3 & 4 |  |
| Explore Values, Operationalize and Learn, and eValuate Efficacy (Peterson et al.) | Stages 3 & 4 |  |
| Extended Parallel Process Model (EPPM) (Witte) | Stages 3 & 4 |  |
| Goal Setting Theory (Locke & Latham) | Stages 3 & 4 |  |
| Health Action Process Approach (HAPA) (Schwarzer) | Stages 3 & 4 |  |
| Health Behavior Framework | Stages 3 & 4 |  |
| Health Belief Model (Rosenstock) | Stages 3 & 4 |  |
| Health Promotion Model (Pender et al.) | Stages 3 & 4 |  |
| Hierarchical Model of Intrinsic and Extrinsic Motivation (Vallerand) | Stages 3 & 4 |  |
| I-Change Model (De Vries et al.) | Stages 3 & 4 |  |
| Implementation Intentions (Gollwitzer) | Stages 3 & 4 |  |
| Information processing model | Stages 3 & 4 |  |
| Information-Motivation-Behavioural Skills Model (Fisher & Fisher) | Stages 3 & 4 |  |
| Interorganizational Relations Theory | Stages 3 & 4 |  |
| Model for Accelerating Improvement (Associates in Process Improvement) | Stages 3 & 4 |  |
| Normalization Process Theory (May) | Stages 3 & 4 |  |
| Operant Learning Theory (Skinner) | Stages 3 & 4 |  |
| Organizational Model of Innovation Implementation (Helfrich) | Stages 3 & 4 |  |
| Partnership Functioning Model (Lasker & Weiss) | Stages 3 & 4 |  |
| PEN-3 (Airhihenbuwa) | Stages 3 & 4 |  |
| Personal Construct Theory (Kelly) | Stages 3 & 4 |  |
| Plan-Do-Study-Act (PDSA) Cycles (Deming) | Stages 3 & 4 |  |
| PPRNet-TRIP Quality Improvement Model (Feifer & Ornstein) | Stages 3 & 4 |  |
| Practical Robust Implementation and Sustainability Model (Feldstein & Glasgow) | Stages 3 & 4 |  |
| Practice change and development model | Stages 3 & 4 |  |
| Precaution Adoption Process Model (PAPM) (Weinstein & Sandman) | Stages 3 & 4 |  |
| Priming theory | Stages 3 & 4 |  |
| Protection Motivation Theory (Rogers) | Stages 3 & 4 |  |
| Self-Affirmation Theory (Steele) | Stages 3 & 4 |  |
| Self-Determination Theory (Deci & Ryan) | Stages 3 & 4 |  |
| Self-Efficacy Theory (Bandura) | Stages 3 & 4 |  |
| Self-Regulation Model of Health and Illness Behaviour (Leventhal et al.) | Stages 3 & 4 |  |
| Self-regulation Theory | Stages 3 & 4 |  |
| Social Comparison Theory | Stages 3 & 4 |  |
| Social Problem-Solving Model (Ewart) | Stages 3 & 4 |  |
| Sociocultural explanatory theory | Stages 3 & 4 |  |
| Theoretical Domains Framework (TDF) (Michie) | Stages 3 & 4 |  |
| Theory of meaningul learning | Stages 3 & 4 |  |
| Theory of Planned Behavior (Ajzen & Fishbein) | Stages 3 & 4 |  |
| Transactional Stress and Coping Model (Lazarus & Folkman) | Stages 3 & 4 |  |
| Transtheoretical Model of Behaviour Change (Prochaska & DiClemente) | Stages 3 & 4 |  |
| Diffusion of Innovations (Rogers) | Stage 4 |  |
| Diffusion of Innovations in Health Service Organizations (Greenhalgh) | Stage 4 |  |
| Core Steps for Effective Implementation (WHO) | Stage 4 |  |
| Development Strategy (Ashford & Patkar) | Stage 4 |  |
| Functional Triad (Fogg) | Stage 4 |  |
| Kirkpatrick's Evaluation Model (Kirkpatrick) | Stage 4 |  |
| NIHR Collaboration for Leadership in Applied Health Research and Care Approach to Implementation | Stage 4 |  |
| Adherence model | N/A | Removed, unretrievable |
| Behavioural Ecological Model of AIDS Prevention | N/A | Removed, topic too specific |
| Cognitive behavioural model of relapse prevention | N/A | Removed, topic too specific |
| Communication Infrastructure Theory | N/A | Removed, topic too specific |
| Diffusion of health promotion innovations | N/A | Removed, unretrievable |
| Framework for cultural sensitivity in health promotion and substance use programs | N/A | Removed, topic too specific |
| Framework for Evidence-Informed Yoga Programs in Oncology | N/A | Removed, topic too specific |
| Health communication theory | N/A | Removed, unretrievable |
| Health education theory | N/A | Removed, unretrievable |
| Integrated mobile ecological model for the promotion of physical activity | N/A | Removed, topic too specific |
| Integrated social cognitive framework | N/A | Removed, unretrievable |
| Knowledge-to-Action Framework (Knowledge-to-Action Model) | N/A | Removed, model is basis of mapping |
| Language expectancy theory | N/A | Removed, topic too specific |
| Learning strategy of exploration, conceptualization, and application (ECA) | N/A | Removed, unretrievable |
| Natural helper (Lay Health Advisor) Intervention Model of Change | N/A | Removed, topic too specific |
| Problem Behaviour Theory | N/A | Removed, topic too specific |
| Program-Planning Model (Kreuter) | N/A | Removed, duplicate to PRECEDE-PROCEED |
| Relapse Prevention Theory | N/A | Removed, topic too specific |
| Self-management thory | N/A | Removed, topic too specific |
| Social Marketing Framework | N/A | Removed, unretrievable |
| Social Normative theory | N/A | Removed, unretrievable |
| Social support theory | N/A | Removed, unretrievable |
| Supply and demand framework | N/A | Removed, unretrievable |
| Theoretical framework related to the work of Grol and Wensing | N/A | Removed, unretrievable |
| Western Australia Health Network (Communities of Practice) approach | N/A | Removed, topic too specific |
| Theory of Implementation Intentions (Gollwitzer) | N/A | Removed, duplicate to Implementation Intentions |
