# Supplementary material for: Selecting implementation models, theories, and frameworks in which to integrate intersectional approaches
Source: BMC Med Res Methodol. 2022 Aug 4;22:212. doi: 10.1186/s12874-022-01682-x (PMC9351159; doi:10.1186/s12874-022-01682-x)
Supplement: Supplementary file 3 — Additional file 3. Project Limitations Statement. [file 12874_2022_1682_MOESM3_ESM.docx]

Project Limitations Statement

We acknowledge that the work of our Canadian Institutes of Health Research (CIHR)-funded team grant was conducted on unceded lands that were the traditional territories of many people, including the Algonquin, Cree, Dakota, Dene, Huron-Wendat, Mississaugas of the Credit River, and the Musqueam Peoples, and on the homeland of the Métis Nation. We acknowledge the harms of the past. We are grateful for the generous opportunities to conduct work and share knowledge on these lands.

In 2017, the CIHR launched an opportunity for team grants in gender and KT. This opportunity (sponsored by the Institute of Gender and Health) was developed to recognize that the field of KT had yet to thoughtfully integrate gender into its research agenda. The objectives of the CIHR team grant competition were to generate evidence about whether applying sex- and gender-based analysis to KT interventions involving human participants improves effectiveness, thereby contributing to improved health outcomes; contribute to a broader knowledge base on how to effectively and appropriately integrate gender into KT interventions; and facilitate the consideration and development of gender-transformative approaches in KT interventions.

In response to this call, we submitted a grant aimed at helping KT intervention developers use an intersectional approach when designing and implementing interventions to address the needs of older adults. We received feedback from the CIHR peer review committee that substantial concern was raised about our focus on intersectionality. In particular, the Scientific Officer’s notes described that the focus on intersectionality would dilute the focus on gender and needed to be reconsidered. A meeting was subsequently held with the successfully funded team and this issue was raised again. We acknowledge the limitation that our intersectional approach comes at the expense of a minimized focus on gender. However, because intersecting categories, such as gender and age, are experienced together, we ultimately elected to use an intersectional approach as it encapsulates the lived experience of those we aim to impact.

A more significant limitation of our work is that we did not include First Nations, Inuit, and Métis community members in the grant proposal. As such, their needs and perspectives were not included in the research grant and, consequently, funded activities. Our team did not have established relationships or expertise in this area and as such, we felt it was inappropriate for our team to work on a grant in this area.

We strongly believe that consideration of gender and KT for Indigenous peoples should be a primary focus of a distinct team grant.

There are established best practices for community engagement with First Nations, Inuit, and Métis Peoples that begin with principles of collaboration, which take time to develop and must not be tokenistic. The principles for collaboration should ensure authentic engagement, shared respect, trust, and commitment to ensure long-term, mutually empowered relationships. These principles should also ensure that the research-related priorities meet the needs, perspectives, and expectations of the First Nations, Inuit, and Métis Peoples. Indigenous peoples have a long history of conducting research, and this tradition continues today with many Indigenous healers and scholars leading research in various areas. Indeed, there are many Indigenous scholars working in the KT field.

Because the team’s work did not include First Nations, Inuit, and Métis Peoples and involve adhering to the principles that guide their engagement in research, the needs and considerations of these Peoples were not included in the work conducted in this team grant. As such, anyone who is considering using the outputs of this team grant needs to know that they cannot be broadly applied to these Peoples and there may be other more culturally appropriate models/theories/frameworks that are useful to consider. Similarly, because this research focused on older adults (and in particular, chronic disease management in older adults) it does not apply to children and youth.

We believe that any KT intervention work needs to begin with engaging the appropriate community and is only applicable when those communities are engaged throughout the research enterprise. Moreover, intersectionality involves deep immersion in the lived experiences and priorities of those communities. As a result, KT work requires immersive work with various populations and not just key informants to ensure the work meets the needs of the relevant populations.

We thank and acknowledge Dr. Lisa Richardson, Co-Lead, Indigenous Health Education, Faculty of Medicine, University of Toronto, for her time and expertise in reviewing this statement.
