# Supplementary material for: Selecting implementation models, theories, and frameworks in which to integrate intersectional approaches
Source: BMC Med Res Methodol. 2022 Aug 4;22:212. doi: 10.1186/s12874-022-01682-x (PMC9351159; doi:10.1186/s12874-022-01682-x)
Supplement: Supplementary file 5 — Additional file 5. Intersecting Categories Survey. [file 12874_2022_1682_MOESM5_ESM.docx]

# Intersecting Categories Survey

Thank you for your contributions to the Intersectionality and Knowledge Translation research study!

The Canadian Institute of Health Research (CIHR) has funded this project and has requested that we specifically measure intersecting categories of all participants and research team members. Please note that you may skip any of these questions if you prefer not to answer them.

All of this information will be presented in an aggregate fashion to preserve confidentiality.

Section 1: Age

1. What is your date of birth? (Adapted from Equality Challenge Unit, 2018)

- (MM/YYYY)
- Prefer not to answer

Section 2: Disability Status

A person with a disability is someone who has a long-term or recurring physical, mental, sensory, psychiatric or learning disability and considers oneself to be disadvantaged by reason of that disability, or believes that society is likely to consider them to be disadvantaged by reason of that disability. A person with a disability may also be someone whose functional limitations owing to their disability have been accommodated in their environment. (Adapted from University of Toronto Department of Medicine Survey, n.d.)

2. Whether or not it affects your day-to-day life, are you a person with a disability? (Adapted from University of Toronto Department of Medicine Survey, n.d.)

Please check ONE only.

- Yes
- No
- Not sure
- Prefer not to answer

The use of 'D/deaf' incorporates those who identify as audiologically deaf and those who are deaf and identify as part of a social and cultural community of deaf people. (Adapted from Equality Challenge Unit, n.d.)

3. Do you have an impairment, health condition or **learning difference that has a substantial or long term impact on your ability to carry out day to day activities? (check all that apply) (Adapted from Equality Challenge Unit, n.d.)**

- No known impairment, health condition or learning difference
- A long standing illness or health condition such as cancer, HIV, diabetes, chronic heart disease, or epilepsy
- A mental health difficulty, such as depression, schizophrenia or anxiety disorder
- A physical impairment or mobility issues, such as difficulty using your arms or using a wheelchair or crutches
- A social/communication impairment such as a speech and language impairment or Asperger’s syndrome/other autistic spectrum disorder
- A specific learning difficulty such as dyslexia, dyspraxia or AD(H)D
- Blind or have a visual impairment uncorrected by glasses
- D/deaf or have a hearing impairment
- An impairment, health condition or learning difference that is not listed above (specify if you wish)
- Prefer not to answer

Section 3: Racial/Ethnic Identity

In Canada, a person of colour or a member of a visible minority group is defined as someone (other than an Indigenous Person) who self-identifies as non-white in colour, regardless of birthplace or citizenship. Members of ethnic or national groups (such as Portuguese, Italian, Greek, etc.) are not considered to be racially visible unless they also meet the criteria above. (Adapted from University of Toronto Department of Medicine Survey, n.d.)

4. Which of the following best describes your race or ethnic group? (Adapted from Toronto Health Equity, 2019; Time, 2018)

Please check ALL that apply.

- Indigenous (e.g., First Nations, Inuit, Métis person from any country)
- Black – African (e.g., Ghanaian, Kenyan, Somali)
- Black – Caribbean (e.g., Barbadian, Jamacian)
- Black – North American (e.g., Canadian, American)
- Latinx – (e.g., Argentinean, Chilean, Salvadorian)
- East Asian (e.g., Chinese, Japanese, Korean, etc.)
- South Asian (e.g., Indian, Pakistani, Sri Lankan, East Indian from Guyana, etc.)
- Southeast Asian (e.g., Filipino, Cambodian, Indonesian, Laotian, Vietnamese, Thai, etc.)
- West Asian (e.g., Iranian, Iraqi, Persian, etc.)
- Central Asian (e.g., Kazakh, Afghan, Tajik, etc.)
- Middle Eastern (e.g., Egyptian, Iranian, Lebanese)
- White / Caucasian – European (e.g., English, Italian, Portuguese, Russian)
- White / Caucasian – North American (e.g., Canadian, American)
- Mixed heritage (e.g., Black- African & White – North American) (Please specify)
- Other (please specify) : __________________________
- Prefer not to answer

Indigenous is a term used to describe the original inhabitants of Canada and their descendants. Indigenous people in Canada include First Nations, Inuit and Métis people. (Adapted from University of Toronto Department of Medicine Survey, n.d.)

5. Are you an Indigenous person? (Adapted from University of Toronto Department of Medicine Survey, n.d.; Aboriginal Peoples Survey, 2017)

Please check ONE only.

- No
- First Nations
- Métis
- Inuit
- Prefer not to answer

Section 4: Gender Identity (Adapted from Bauer et al., 2017)

6. What sex were you assigned at birth, meaning on your original birth certificate?

- Male
- Female
- Prefer not to answer

7. Which best describes your current gender identity?

- Male
- Female
- Indigenous or other cultural gender minority identity (e.g., two-spirit)
- Something else (e.g., gender fluid, non-binary) : ___________________________
- Prefer not to answer

“The third question may be asked only of those who indicated a current gender identity different than their birth-assigned sex. If so, it can be forward-filled to code cisgender participants as living in their identified (and birth-assigned) sex/gender.” (Bauer et al., 2017)

8. What gender do you currently live as in your day-to-day life?

- Male
- Female
- Sometimes male, sometimes female
- Something other than male or female
- Prefer not to answer

Section 5: Sexual Orientation (Adapted from Toronto Health Equity, 2019)

9. What is your sexual orientation?

- Heterosexual
- Gay
- Lesbian
- Bisexual
- Queer (a term used by people who do not follow common sexual orientations)
- Two-Spirit (a term used by Indigenous people)
- Prefer to self-describe (please specify)
- Do not know
- Prefer not to answer

Section 6: Socioeconomic Status (Adapted from Toronto Health Equity, 2019)

10. What was your total family income before taxes last year?

- $0 - $29.999
- $30,000 – $59,999
- $60,000 - $89,999
- $90,000 - $119,999
- $120,000 - $149,999
- $150,000 or more
- Do not know
- Prefer not to answer

Section 7: Social Capital (Adapted from Adler, M., & Stewart, J., 2007)

11. Think of the ladder below as showing where people stand in their communities. People define ‘community’ and different ways. Please define ‘community’ in whatever way is most meaningful to you. At the top of the ladder are the people who have the highest standing in their community. At the bottom of the ladder are people who have the lowest standing in their community.

Where would you place yourself on this ladder? Place an X on the ladder rung where you think you stand at this time of your life relative to other people in your community.

|  |
| --- |
|  |
|  |
|  |
|  |
|  |
|  |
|  |
|  |
|  |
|  |

- Prefer not to answer

Section 8: Housing (Adapted from Toronto Health Equity, 2019)

12. What type of housing do you live in?

- Own Home
- Renting Home
- Boarding Home
- Correctional Facility
- Homeless/on street
- Group Home
- Shelter/Hostel
- Supportive Housing
- Other (specify): ___________________
- Do not know
- Prefer not to answer

Section 9: Place of residence (Adapted from Statistics Canada, 2018)

13. Which of the following best describes your place of residence?

- Rural area, with a population less than 1,000
- Small population center, with a population between 1,000 and 29,999
- Medium population center, with a population between 30,000 and 99,999
- Large urban population center, with a population of 100,000 or more
- Prefer not to answer

Section 10: Language

14. What language(s) do you speak (e.g., fill in the blank with ‘English’)?

- Fill in the blank: ________ (Language 1)
- Fill in the blank: ________ (Language 2)
- Fill in the blank: ________ (Language 3)
- Fill in the blank: ________ (Language 4)
- Prefer not to answer

15. What is your degree of verbal and written fluency for the language(s) you speak? Please fill in the box below with the rating below for your verbal and written fluency for the language(s) you speak (see previous question).

1. Very weak, 2. Weak, 3. Slightly Weak, 4. Average, 5. Slightly Strong, 6. Strong, 7. Very Strong

| Language 1 – Verbal |  |
| --- | --- |
| Language 1 – Written |  |
| Language 2 – Verbal |  |
| Language 2 – Written |  |
| Language 3 – Verbal |  |
| Language 3 – Written |  |
| Language 4 – Verbal |  |
| Language 4 – Written |  |

- Prefer not to answer

Section 11: Education (Adapted from Survey Monkey, n.d.)

16. What is the highest level of education you have completed? Check all that apply to your current situation.

- Did not attend school
- Grade 6
- Grade 8
- Graduated from high school
- Some CÉGEP
- Completed CÉGEP (e.g., Diploma of College Studies)
- Some college
- Completed College diploma (e.g., Certificate in Business, Diploma in Human Resources)
- Some undergraduate university
- Completed Bachelor’s degree (e.g., Bachelor of Arts, Bachelor of Science)
- Some graduate school (university)
- Completed Master’s degree (e.g., Masters in Public Health, Masters in Marketing)
- Some professional school
- Completed professional school (e.g., Doctor of Medicine, Juris Doctor)
- Some doctorate
- Completed doctorate (e.g., Doctor of Philosophy)
- Other (please specify): ___________________
- Prefer not to answer

Section 12: Employment (Adapted from UK Data Service, n.d.)

17. Which of the following best describes your current employment status? Check all that apply to your current situation.

- Full-time employed
- Part-time employed
- Temporarily laid off
- Not employed for pay
- Retired
- Permanently living with a disability
- Caregiver (e.g., children, elderly)
- Homemaker
- Full-time student
- Part-time student
- Other (please specify): ___________________
- Prefer not to answer

Section 13: Caring responsibilities (Adapted from Equality Challenge Unit, n.d.)

18. Do you have any caring responsibilities? Check all that apply to your current situation.

- None
- Primary carer of a child or children (under 18 years)
- Primary carer of a child or children with a disability
- Primary carer or assistant for an adult (18 years and over) with a disability
- Primary carer or assistant for an older person or people (65 years and over)
- Co-carer (carries out main caring role with another person)
- Secondary carer (another person carries out main caring role)
- Prefer not to answer

Section 14: Marriage/civil partnership (Adapted from Equality Challenge Unit, n.d.)

19. Are you currently?

- Cohabiting
- Divorced or civil partnership dissolved
- In a civil partnership
- Married
- Separated (but still legally married or in a civil partnership)
- Single (never married or never in a civil partnership)
- Widowed or a surviving partner from a civil partnership
- Prefer not to answer

Section 15: Religion or Spiritual Affliation (Adapted from Toronto Health Equity, 2019)

20. What is your religious or spiritual affiliation? Check all that apply to your current situation.

- Christian Orthodox
- Protestant
- Roman Catholic
- Christian, not included elsewhere on this list
- Animism or Shamanism
- Atheism
- Baha’i Faith
- Buddhism
- Confucianism
- Hinduism
- Jainism
- Judaism
- Islam
- Indigenous Spirituality
- Rastafarianism
- Sikhism
- Spiritual
- Unitarianism
- Pagan
- Zoroastrianism
- Other (Please specify): ___________________
- Do not know
- I do not have a religious or spiritual affiliation
- Prefer not to answer

Section 16: Taking leave (Adapted from Equality Challenge Unit, n.d.)

21. Have you taken any of the following types of leave within the past year? Check all that apply.

- Emergency leave
- Bereavement leave
- Medical leave
- Family Medical leave
- Maternity/Paternity leave
- Adoption leave
- Shared parental leave
- Other type of leave: ___________________
- Prefer not to answer

If you have any questions, concerns, or technical difficulties, please do not hesitate to contact Danielle Kasperavicius ([kasperavicid@smh.ca](mailto:kasperavicid@smh.ca) ; 416 864-6060 ext. 76224).

References

Adler, M., & Stewart, J. (2007, March). The MacArthur Scale of Subjective Social Status [Info Page]. Retrieved January 22, 2019, from <https://macses.ucsf.edu/research/psychosocial/subjective.php>

Bauer, G. R., Braimoh, J., Scheim, A. I., & Dharma, C. (2017). Transgender-inclusive measures of sex/gender for population surveys: Mixed-methods evaluation and recommendations. *PLOS ONE*, *12*(5), e0178043. <https://doi.org/10.1371/journal.pone.0178043>

CBC News. (2016, September 22). Indigenous or Aboriginal: Which is correct? [News]. Retrieved January 21, 2019, from <https://www.cbc.ca/news/indigenous/indigenous-aboriginal-which-is-correct-1.3771433>

Equality Challenge Unit. (n.d.). Monitoring questions [Info Page]. Retrieved January 21, 2019, from <https://www.ecu.ac.uk/guidance-resources/using-data-and-evidence/monitoring-questions/>

Equality Challenge Unit. (2018). Guidance on gathering staff equality data for colleges [Info Page]. Retrieved January 21, 2019, from <https://www.ecu.ac.uk/publications/guidance-gathering-staff-equality-data-colleges/>

Government of Canada, S. C. (2017, January 10). Aboriginal Peoples Survey 2017 - Economic participation [Info Page].. Retrieved January 21, 2019, from <http://www23.statcan.gc.ca/imdb/p3Instr.pl?Function=assembleInstr&lang=en&Item_Id=331955#qb332233>

Scottish Government, S. A. H. (2015, November 23). Scottish Surveys Core Questions [Info Page]. Retrieved January 21, 2019, from <http://www2.gov.scot/Topics/Statistics/About/Surveys/SSCQ>

Statistics Canada. (2018, May 22). Level of Education Demographics Survey Question. Retrieved January 23, 2019, from <https://www.surveymonkey.com/mp/education-demographics-survey-template/>

Survey Monkey (n.d.). Level of Education Demographics Survey Question. Retrieved January 23, 2019, from <https://www.surveymonkey.com/mp/education-demographics-survey-template/>

Time. (2018, April 2). Why “Latinx” Is Succeeding While Other Gender-Neutral Terms Fail to Catch On. [News]. Retrieved January 23, 2019, from <http://time.com/5191804/latinx-definition-meaning-latino-hispanic-gender-neutral/>

Toronto Health Equity. (2019). Demographic Questions [Info Page]. Retrieved January 21, 2019, from <http://torontohealthequity.ca/demographic-questions/>

UBC Indigenous Foundations. (n.d.). Terminology [Blog Post]. Retrieved January 23, 2019, from <https://indigenousfoundations.arts.ubc.ca/terminology/>

UK Data Service. (n.d.). Variable & Question Bank - Public Attitudes to Price Fixing and Cartel Enforcement in Britain, Germany, Italy and the United States, 2014. Retrieved January 23, 2019, from [https://discover.ukdataservice.ac.uk//variables/variable/?id=7885_V265](https://discover.ukdataservice.ac.uk/variables/variable/?id=7885_V265)
